# Effect of TREM-1 blockade and single nucleotide variants in experimental renal injury and kidney transplantation

**DOI:** 10.1038/srep38275

**Published:** 2016-12-08

**Authors:** Alessandra Tammaro, Jesper Kers, Diba Emal, Ingrid Stroo, Gwendoline J. D. Teske, Loes M. Butter, Nike Claessen, Jeffrey Damman, Marc Derive, Gerjan J. Navis, Sandrine Florquin, Jaklien C. Leemans, Mark C. Dessing

**Affiliations:** 1 Department of Pathology, Academic Medical Center, University of Amsterdam, Amsterdam, the Netherlands; 2 Inserm UMR_S1116, Faculté de Médecine de Nancy, Université de Lorraine, Vandoeuvre-les-Nancy, France; 3 Department of Internal Medicine, Division of Nephrology, University Medical Center Groningen, University of Groningen, Groningen, the Netherlands; 4 Department of Pathology, Radboud University Nijmegen Medical Center, Nijmegen, the Netherlands

## Abstract

Renal ischemia reperfusion (IR)-injury induces activation of innate immune response which sustains renal injury and contributes to the development of delayed graft function (DGF). Triggering receptor expressed on myeloid cells-1 (TREM-1) is a pro-inflammatory evolutionary conserved pattern recognition receptor expressed on a variety of innate immune cells. TREM-1 expression increases following acute and chronic renal injury. However, the function of TREM-1 in renal IR is still unclear. Here, we investigated expression and function of TREM-1 in a murine model of renal IR using different TREM-1 inhibitors: LP17, LR12 and TREM-1 fusion protein. In a human study, we analyzed the association of non-synonymous single nucleotide variants in the *TREM1* gene in a cohort comprising 1263 matching donors and recipients with post-transplant outcomes, including DGF. Our findings demonstrated that, following murine IR, renal TREM-1 expression increased due to the influx of *Trem1* mRNA expressing cells detected by *in situ* hybridization. However, TREM-1 interventions by means of LP17, LR12 and TREM-1 fusion protein did not ameliorate IR-induced injury. In the human renal transplant cohort, donor and recipient *TREM1* gene variant p.Thr25Ser was not associated with DGF, nor with biopsy-proven rejection or death-censored graft failure. We conclude that TREM-1 does not play a major role during experimental renal IR and after kidney transplantation.

Kidney transplantation is at present the most optimal renal replacement therapy for patients with end-stage renal disease (ESRD). Following transplantation, renal ischemia reperfusion (IR)-induced injury is a major cause of delayed graft function (DGF). DGF is associated with an increased risk for acute rejection and decreased survival of the allograft^1^,^2^. Innate immunity plays an important role in the mechanism underlying IR-induced injury. Following kidney injury, damage-associated molecular patterns (DAMPs) are released from necrotic cells and recognized by pattern recognition receptors (PRRs) that include toll like receptors (TLRs). Activation of TLRs is known to induce inflammation that affects renal function following IR^3^,^4^. Over the past decade, an additional family of innate immune receptors has been identified: the triggering receptors expressed on myeloid cells (TREMs)^5^,^6^,^7^. TREM-1 is mainly expressed on granulocytes and monocyte/macrophages in mouse and human^8^. TREM-1 is an activating receptor, which associates with its adaptor molecule TYRO protein tyrosine kinase-binding protein (TYROBP) to induce cytokine production^5^,^6^,^7^. Besides from activating its own intracellular pathway, TREM-1 synergizes with diverse TLRs, leading to an amplified inflammatory responses^5^,^6^,^7^,^8^. Most of the studies addressing the pathogenic role of TREM-1 have been performed in infectious disease models^9^,^10^. The general concept thus far is that TREM-1 is specifically involved in anti-microbial immune responses^11^. Recent evidence, however, has also pointed towards a beneficial effect of TREM-1 inhibition during sterile inflammation, like IR^12^,^13^. Murine studies have shown that TREM-1 expression increases upon chronic obstructive nephropathy and renal IR^14^,^15^,^16^. In humans, renal TREM-1 expression has been observed on interstitial cells of patients with obstruction-related hydronephrosis^15^. Blockade of the TREM-1 signaling by a short inhibitory peptide (LP17 and LR12) reduced tissue injury during mesenteric IR and myocardial infarction, emphasizing the potential therapeutic benefit of TREM-1 inhibition in sterile inflammation^12^,^13^. Currently, the treatment of patients with acute kidney injury in the context of DGF is purely supportive, whereas manipulation of innate immunity during necroinflammation might further reduce alloimmune priming, leading to a reduction in rejection. Moreover, genetic variation may also determine the course of graft injury and be linked to the risk of DGF. In the current study we investigated whether TREM-1 could be a potential target during experimental and human renal IR-induced injury.

We therefore investigated (1) the expression and function of TREM-1 in murine renal IR and (2) determined the association between non-synonymous single nucleotide variants (SNVs) in the *TREM1* gene and outcomes following renal transplantation, with a particular interest for the risk to develop DGF.

## Results

### Renal ischemic injury leads to increased TREM-1 expression

**Figure 1:**
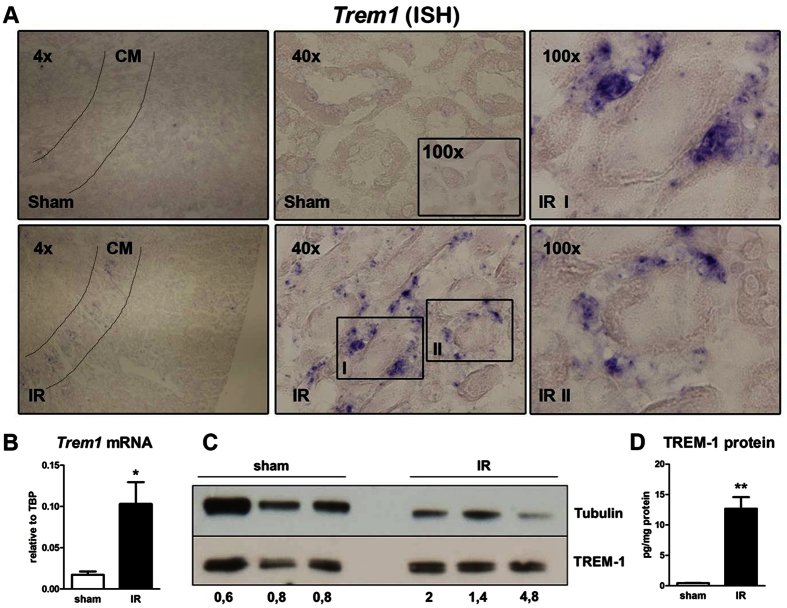
TREM-1 expression is increased in the kidney after IR. *Trem1* transcript expression visualized by using *in situ* hybridization (ISH) in sham and 1 day after bilateral IR. Lines in sham and IR (4× magnification) indicate the cortico-medullary (CM) area. Sham pictures (40×) shows an inset with 100× magnification. From the IR (40×) picture we selected two areas (I and II) to show in higher magnification (100×). Original magnification 4×, 40× and 100× (**A**). Intrarenal *Trem1* transcript quantified by RT-PCR (n = 6–7/group) (**B**) and protein expression as determined by western blot (n = 3) and ELISA (n = 6–7/group) (**C**,**D**). Values are expressed as mean ± SEM. *P < 0.05 vs sham; **P < 0.005 versus sham.

**Figure 2:**
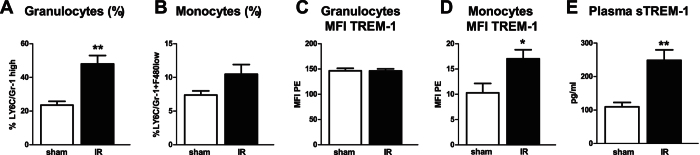
Renal IR increases circulating soluble TREM-1 expression. Percentage of circulating Ly6C/Gr-1 high granulocytes (**A**) and Ly6C/Gr-1+ F4-80 low monocytes (**B**) of total leukocytes, 1 day after IR (the gating strategy can be found as Supplementary Fig. S2). Membrane TREM-1 expression on granulocytes (**C**) and monocytes (**D**) as displayed by mean fluorescence intensity (MFI) (n = 5–8/group). Circulating TREM-1 soluble protein in sham and one day after IR, measured by ELISA (n = 7/group) (**E**). Values are expressed as mean  ± SEM. *P < 0.05 vs sham. **P < 0.005 versus sham.

The S3 segment of the proximal tubules located in the cortico-medullary (CM) area is the most sensitive to ischemic injury^17^. Moreover, the interstitial cells surrounding the ischemic tubules are rich in granulocytes that accumulate in the kidney after reperfusion. Since TREM-1 is expressed on the plasma membrane of granulocytes, we determined renal *Trem1* mRNA expression 24 hours after renal IR. Using *in situ* hybridization, we localized *Trem1* transcript expression in kidney tissues from mice one day after IR. Sham tissues were used as control. *Trem1* mRNA-positive interstitial cells were detected in the CM area, after IR and absent in sham kidney. Noteworthy, baseline or damaged tubular epithelial cells did not stain positive for *Trem1* transcripts (Fig. 1A). Moreover, we quantified renal *Trem1* transcription by RT-PCR (Fig. 1B) and observed an increased expression in IR kidneys compared to sham tissues, which was confirmed on the protein level by western blot and ELISA (Fig. 1C,D). Following IR, inflammatory cells appear in the circulation to subsequently migrate to the site of injury^17^. By FACS analysis, we detected an increased percentage of circulating granulocytes (Fig. 2A) identified as Ly6C/Gr-1 high populations, following IR. Percentage of circulating monocytes (Ly6C/Gr-1 positive-F4-80 low population as shown in Supplementary Fig. S1) instead, were similar between sham and IR mice (Fig. 2B). This suggests that renal *Trem1* mRNA-expressing cells are most likely infiltrating granulocytes. We then checked the surface expression of TREM-1 receptor on circulating granulocytes and monocytes from sham and IR mice. Renal IR leads to up-regulation of TREM-1 receptor on the plasma membrane of circulating monocytes, but not granulocytes (Fig. 2C,D) and also to increased expression of the soluble form of TREM-1 in the plasma (Fig. 2E). These findings indicate that renal IR may modulate the inflammatory response by regulation of TREM-1 surface expression.

### Administration of TREM-1 inhibitors reduces *Trem1* and *Myd88* transcription

Once we established that TREM-1 expression is indeed increased following IR, we aimed to determine the functional role of TREM-1 in renal IR. We tested the hypothesis that blocking the ligand-receptor binding by various strategies could affect renal IR-induced injury. Mice were treated with different types and doses of TREM-1 inhibitors (as indicated in the methods section) and underwent uni- or bilateral clamping of the renal artery. To confirm that the TREM-1 pathway was indeed blocked by the treatment with the various inhibitors, we measured renal *Trem1* mRNA expression in the different groups. The majority of TREM-1 inhibitors led to downregulation of renal *Trem1* transcription (see Supplementary Fig. S2A) as was previously shown^13^. Because there is evidence that TREM-1 activation regulates MYD-88 signaling and hence it controls the extent of the inflammatory response via this signaling pathway^18^,^19^,^20^,^21^, we measured *Myd88* mRNA transcription in renal tissue as downstream readout of TREM-1 blockade^18^. In line with the previous results, we could show that following renal IR, treatment with Fc-TREM1, LR12 and LP17 affect *Myd88* mRNA expression (see Supplementary Fig. S2B), whereas *Trif* transcript levels were relatively unchanged (data not shown)^18^.

### Modulation of TREM-1 function affects renal cytokine and chemokine transcription profiles

**Figure 3:**
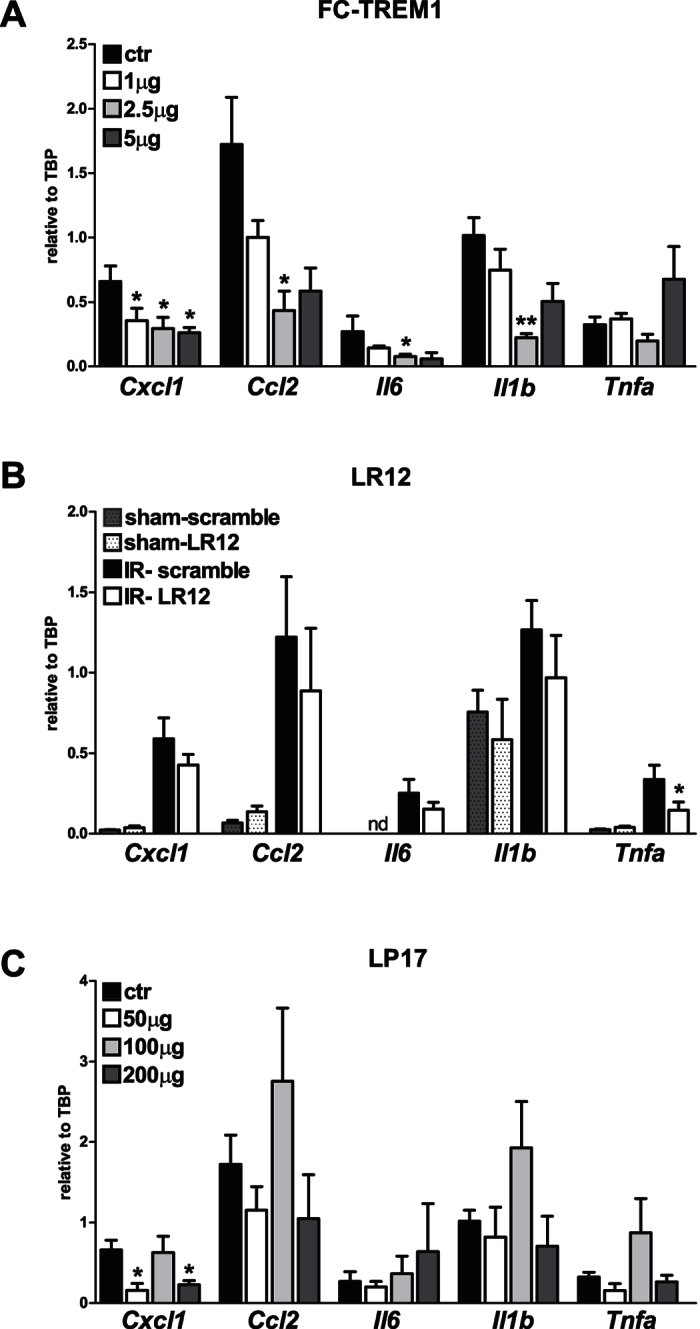
Renal cytokine transcripts are differentially affected by TREM-1 inhibitors following IR. Gene expression of *Cxcl1* (the gene for KC), *Ccl2* the gene for (MCP-1), *Il6*, *Il1b* and *Tnfa* in kidney of mice treated with recombinant Fc-TREM1 (n = 4/group) (**A**). In (**B**) the level of pro-inflammatory transcripts in mice treated with LR12 or scramble protein (n = 6–8/group). In the last panel (**C**) the inflammatory results of LP17 injections (n = 4/group). Values are normalized to the housekeeping gene *Tbp*. Data are expressed as mean ± SEM, *P < 0.05 versus control. **P < 0.005 versus control.

TREM-1 is a well-known amplifier of the inflammatory response, thus we investigated whether TREM-1 blockade may affect renal inflammation. The expression of cytokines and chemokines that play a role during renal inflammation were measured in renal tissues by RT-PCR. The renal inflammatory reaction was mostly dampened by different doses of recombinant TREM-1 pre-treatment. Expression of *Cxcl1* (the gene for KC), *Ccl2* (the gene for MCP-1), *Il6*, *Il1b* and *Tnfa* was impaired compared to renal ischemic tissue receiving the isotype control treatment (Fig. 3A). Next, we investigated the more potent TREM-1 inhibitor LR12^22^. Due to the short half-life of LR12^23^, mice were treated every 8 hours until sacrifice. Sham mice received the same treatment to exclude any detrimental effect due to repeated injections. Unlike Fc-TREM-1, LR12 treatment only downregulated *Tnfa* transcription (Fig. 3B). Finally, we evaluated the function of LP17 peptide, the TREM-1 inhibitor proposed as a therapeutic tool to dampen TREM-1 induced inflammation in several disease settings^12^,^20^,^21^,^22^,^23^,^24^. We found that LP17 affects *Cxcl1* (KC) transcription, but not the transcription of the other inflammatory mediators (Fig. 3C).

We next evaluated the inflammatory mediators on the protein level to check whether the transcript expression was translated into protein. We measured the concentrations of KC, MCP-1, IL-6 and IL-1β protein by ELISA and found that compared to sham mice, KC and IL-1β concentrations were significantly elevated 24 hours after IR. TREM-1 inhibition did not lead to any change in majority of cytokine and chemokine protein levels. The only difference we detected was related to LR12 treatment. We found that IL-1 β was significantly decreased in the LR12-treated mice that underwent IR as compared to their IR controls with levels comparable to the sham treated animals (P = 0.02, Table 1).

### Administration of TREM-1 inhibitors does not prevent the development of renal injury

**Figure 4:**
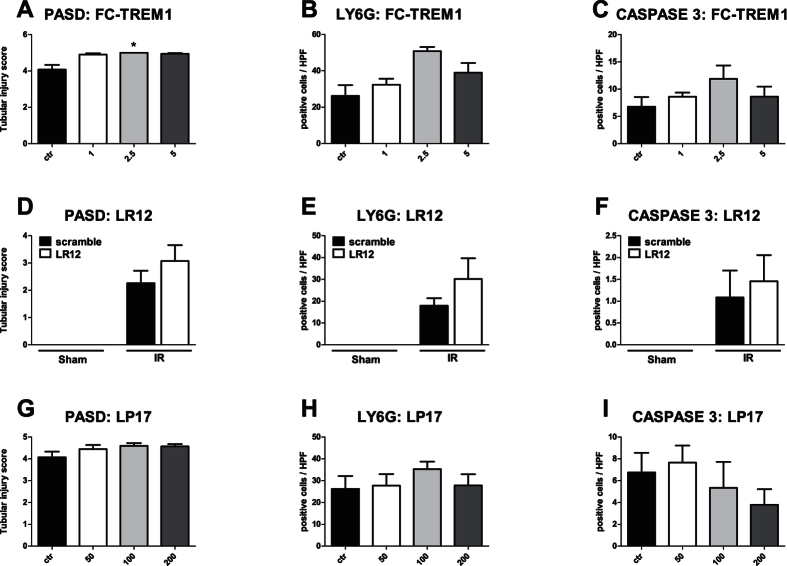
TREM-1 modulation does not prevent unilateral renal IR-induced injury. Renal damage (PAS-D score: **A**,**D**,**G**), influx of granulocytes (Ly6G positive cells/HPF: **B**,**E**,**H**) and tubular apoptosis (cleaved caspase 3 positive tubular epithelial cell/HPF: **C**,**E**,**H**) in mice 24 hours after unilateral IR. In order from the top row, results of Fc-TREM1 (n = 4/group), LR12 (n = 6–8/group) and LP17 treatment (n = 4/group). Data are expressed as mean ± SEM, *P < 0.05 versus control.

Renal IR injury after 24 hours is characterized by tubular injury and infiltration of granulocytes. In unilateral IRI, mice pre-treated with different doses of Fc-TREM1 or matching isotype control did not display a change in renal damage (morphologically by PAS-D staining), infiltration of Ly6G-positive granulocytes and tubular epithelial cell apoptosis (detected by immuno-histochemical staining of cleaved caspase 3) (Fig. 4A–C).

Consistent with the results obtained for Fc-TREM1 treatment, LR12 treatment did not lead to any change in terms of renal damage, granulocyte infiltration or tubular apoptosis compared to mice receiving scramble protein (Fig. 4D–F). The same results were observed for LP17-treated mice (Fig. 4G–I). Renal function as measured by plasma creatinine and urea, due to contralateral filtration, was not affected (data not shown).

Because the potential therapeutic significance of LP17 treatment has been extensively shown by many other groups and especially in sterile inflammation settings^12^,^20^, we tested whether the neutral results obtained were dependent on the degree of damage. Thus we induced a more severe damage model with bilateral renal IR surgery and prior treatment with LP17 or scramble peptide. In the bilateral model, renal dysfunction parameters (plasma creatinine and urea) increased compared to sham mice. LP17 treated mice did not display any significant effect on plasma urea and creatinine concentrations, renal damage score (PAS-D) or inflammation (renal granulocyte influx) compared to control-peptide treated mice (see Supplementary Fig. S3).

Taken together, our experiments indicate that modulation of TREM-1 function in the course of renal IR leads to partial downregulation of the inflammatory response, but this did not significantly affect tubular damage, renal granulocyte influx, tubular apoptosis or renal function.

### Study characteristics and distribution of the *TREM1* SNVs in donors and recipients

We investigated whether SNVs in the human *TREM1* gene were associated with outcome following renal transplantation, with DGF in particular. The cohort characteristics are shown in Table 2. The minor allele frequency of the two non-synonymous SNVs in the *TREM1* gene for donors and recipients were respectively 0.078 and 0.085 for p.Thr25Ser and 0.001 and 0.000 for p.Phe214Leu without statistical differences between donors and recipients (*P* > 0.8 Table 3). Donors and recipients significantly deviated from Hardy-Weinberg equilibrium (*P* = 0.003 and *P* = 0.002 for donors and recipients), but both distributions were not different from the frequency distribution found in the HapMap-CEU population of the 1000 Genomes sequencing data.

### *TREM1* single nucleotide variants do not associate with renal transplant outcomes

DGF occurred in 411/1263 patients (33%), of which 60 resulted in primary non-function and were therefore excluded from analyses. The majority (97%) of patients who experienced DGF were recipient of a deceased donor. For the p.Phe214Leu variant, we acquired insufficiently high MAFs for further analyses. In the full cohort, the *TREM1* p.Thr25Ser variant was not significantly associated with DGF in donors (OR_heterozygosity_ = 1.08, 95% CI = 0.77–1.52, *P* = 1) and recipients (OR_heterozygosity_ = 0.91, 95% CI = 0.64–1.27, *P* = 1). Five renal transplant recipients were homozygous recessive for the *TREM1* variant p.Thr25Ser. None of these patients developed DGF, but due to the small group, statistical analysis was not performed (Supplementary Table S1). On follow-up, 430/1263 (34%) of recipients encountered a first episode of biopsy proven acute rejection. The median (interquartile range) time to rejection was 51 months (1–105 months). Neither donor nor recipient *TREM1* p.Thr25Ser variants associated with the cumulative incidence of biopsy proven acute rejection in donors (HR_heterozygosity_ = 0.72, 95% CI = 0.54–0.96, *P* = 0.15 and HR_heterozygosity_ = 0.81, 95% CI = 0.62–1.07, *P* = 0.84, respectively). Death censored graft failure occured in 215/1263 (17%) at a median (interquartile range) of 5.5 years (2.9–8.7 years) after transplantation.

Corroborating on the results obtained for death censored graft failure and biopsy proven acute rejection, neither donor nor recipient *TREM1* p.Thr25Ser associated with the cumulative incidence of death censored graft failure in donors or recipients (HR_heterozygosity_ = 0.75, 95% CI = 0.50–1.13, *P* = 1 and HR_heterozygosity_ = 0.87, 95% CI = 0.59–1.27, *P* = 1, respectively). When we stratified patients for donation type (DBD or DCD), similar results were obtained (Table 4). In conclusion, we did not acquire evidence for a potential effect of SNVs in the *TREM1* gene for post-transplant renal outcomes, including DGF.

## Discussion

Infiltrating inflammatory cells contribute to the development of acute kidney injury following IR, which increases the risk of DGF^17^. Increasing evidence suggest that TREM-1 plays an essential role in modulating the innate immune response in (non) sterile acute and chronic inflammation^10^,^11^,^13^,^21^. TREM-1 enhances the inflammatory response through synergism with TLR pathway signaling^7^. The contribution of TLRs in sterile renal injury has been broadly investigated in mice and humans^4^,^25^. However, the role of TREM-1 herein is still unclear. Interestingly, blockade of TREM-1 activation by synthetic analogues reduced pathology in mesenteric IR and myocardial infarction^12^,^13^. In the current study we showed that synthetic TREM-1 inhibition in murine renal IR-induced injury does not appear to be a therapeutic target to prevent injury and/or improve function. Consistent with the results of the experimental study, we found no association of the p.Thr25Ser heterozygous variant with renal outcome following kidney transplantation.

Our findings demonstrated that renal IR induced a significant increase in *Trem1* transcripts in renal tissue. This up-regulation was in line with the increased number of circulating granulocytes, well known to express TREM-1 and to accumulate in the kidney tissue as early as 30 minutes after reperfusion^26^. We speculate that *Trem1* mRNA expressing cells, as detected by *in situ* hybridization, are most probably infiltrating granulocytes. Blood monocytes are recruited into the kidney in response to a gradient of chemokines, especially monocyte chemoattractant protein-1 (MCP-1), which peaks in IR kidney later than 24 hours^27^. Thus, no differences were observed in the number of circulating monocytes between sham and IR mice, probably because of timing of the necropsies after reperfusion. However, we found that IR leads to up-regulation of TREM-1 surface expression on circulating monocytes. Blood monocytes infiltrate inflamed kidney tissues and differentiate into macrophages and dendritic cells^17^. Thus, our data would suggest a role for TREM-1 in macrophage-mediated renal injury. This hypothesis has previously been discussed in the literature^14^. Together with an increased membrane receptor expression, we detected increased levels of plasma soluble TREM-1. The soluble protein can originate from proteolytic cleavage from monocytes^28^ or new splice variant synthesis^29^. The latter hypothesis is conceivable with our results.

The increased circulating sTREM-1 protein concentration following IR is in line with other studies showing that the soluble protein can be detected in plasma, fluids and urine during infection and inflammation^30^,^31^,^32^,^33^. Functionally, the soluble protein acts as a counter regulatory molecule by scavenging available TREM-1 ligand thus preventing further amplification of the inflammatory signal. Nevertheless, in renal IR, the significance of this increased concentration of sTREM-1 appears to be dispensable in modulating the inflammatory response. Several studies have shown that treatment with TREM-1 inhibitors represents a therapeutic strategy to prevent excessive inflammation resulting in decreased pathology and mortality. The inhibitory peptide LP17 was shown to successfully reduce the inflammatory responses in the context of infection, mesenteric IR-induced injury, hemorrhagic shock, colitis, autoimmune arthritis and acute pancreatitis^12^,^20^,^34^,^35^,^36^,^37^,^38^,^39^. In addition, TREM-1 fusion protein and LR12 reduced the disease severity in sepsis and in models of acute myocardial infarction^13^,^23^,^24^,^40^,^41^,^42^. These previous studies and the significantly elevated TREM-1 expression during renal IR would suggest a potential benefit from TREM-1 interventions to reduce renal IR-induced injury. In our study, we relied on the administration of commercial available TREM-1 synthetic inhibitors. We showed that treatment with different inhibitors is able to modulate *Trem1* mRNA expression and more interestingly, TREM-1 blocking resulted in a decreased *Myd88* transcription, as previously showed by^18^. Nevertheless, the reduced transcription of pro-inflammatory markers as measured by RT-PCR as a result of decreased *Myd88* expression, was not mirrored by a significant decrease in protein concentrations. Because of discrepancy between mRNA and protein, we believe that in this study, given the final readout, protein levels are biological relevant. Indeed, in line with these findings and contrary to the above-mentioned studies addressing the pathogenic role of TREM-1, our results have shown that interventions that modulate TREM-1 function did not prevent renal IR.

Supporting our results, Campanholle *et al*. in a recent study have shown that administration of TREM-1 fusion protein did not have any effect during recovery from renal IR^14^. However, the authors aimed to answer a different research question: unraveling the therapeutic potential of TREM-1 in macrophage activation and fibrosis, a later stage of renal inflammation compared to our study. Granulocytes and macrophages have a different temporal distribution and play a distinct role in renal IR^27^. Thus, our results are not fully comparable with those of Campanholle *et al*., but provide more evidence that blocking TREM-1 signaling at the early stage of renal IR does not represent a suitable therapeutic target. Thus far it appears that TREM-1 blocking agents provide controversial findings in sterile inflammatory disorders. Although we observed TREM-1 up-regulation following renal IR, this functional study showed that TREM-1 does not play a pivotal role in granulocyte- and monocyte-induced renal inflammation. We speculate that divergent results obtained by TREM-1 inhibitors might be dependent on affinity ligand availability. In our model of sterile renal injury, we detected *Trem1* mRNA expression in inflammatory cells rather than tubular cells. Previous study emphasized that in sterile ischemic injury, renal parenchyma-associated TLR-2 and TLR-4, rather than inflammatory cell-associated TLRs contribute to renal dysfunction^3^,^4^,^43^. The neutral results observed in terms of renal outcome could be related to the dispensable role of innate immune receptor expression on inflammatory cells in this disease setting. Additionally, renal DAMPs that are released by damaged parenchymal cells, might have higher affinity for TLRs as compared to TREM-1. In the presence of a low abundance of or a low affinity for TREM-1 ligand, TLR signaling might be dominant, thus the inflammatory response in renal IR would then be TREM-1 independent or the TREM-1 signaling pathway would be of low significance. However, it cannot be excluded that the ligands, through redundancy, could activate the inflammatory cells through an alternative receptor, for instance TREM-3, which most likely can compensate for TREM-1 blockade because of comparable function^14^. In other models of sterile inflammation where TREM-1 inhibition had a beneficial effect, the amplification of the inflammatory response might have been TREM-1 dependent.

Over the past years, soluble TREM-1 has gained interest as biomarker of several human disease settings including renal dysfunction^29^,^44^,^45^. Since then, also genetic studies that aimed at finding an association between variants in the *TREM1* gene and outcomes in various clinical settings were conducted, however with conflicting (or disease-specific) results. SNVs in the *TREM1* gene were shown to associate with intestinal Behcet’s disease, pneumonia in burn patients, risk for coronary artery disease but not with inflammatory bowel diseases or infectious endocarditis^46^,^47^,^48^,^49^,^50^. There is no consensus on the association of *TREM1* variants with (the outcome after) sepsis with Chen *et al*.^51^ indicating no association in a Chinese population comprising 175 patients with sepsis and Su *et al*. describing a significant correlation with the incidence of sepsis in 80 Chinese patients^48^. Interestingly, Su *et al*. did not describe an association between the p.Thr25Ser variant of *TREM1* with the (dynamic) concentrations of sTREM-1^48^, which questions whether there truly is a biochemical consequence of this non-synonymous variant. To the best of our knowledge, the current study is the first on *TREM1* genetic variants in renal transplant patients. The lack of association between the *TREM1* p.Thr25Ser heterozygous variant and renal outcomes with DGF in particular is in line with the lack of beneficial potential in the preclinical mouse studies of IR-induced injury. However, even though this was a large study, we cannot exclude that patients with homozygosity for the p.Thr25Ser variant are in fact protected from IR-induced injury after transplantation (N = 5, Supplementary Table S1).

From these studies we conclude that (1) TREM-1 increases during experimental renal IR due to *Trem1* gene transcription by infiltrating leukocytes, (2) various interventions aiming at modulating the function of murine TREM-1 downregulated the inflammatory response, but did not prevent renal damage, leukocyte influx or organ dysfunction and (3) genetic variants in the *TREM1* gene do not associate with the development of delayed graft function, biopsy proven acute rejection or subsequent graft failure. Taken together, these experiments question TREM-1 as a potential target of therapy in these particular disease settings.

## Materials and Methods

### Surgical procedure of murine ischemia reperfusion

Pathogen-free 8-to 12 week-old male C57BL/6 WT were purchased from Charles River Laboratories. The Animal Care and Use Committee of the University of Amsterdam approved all the experiments in compliance with the ARRIVE guidelines (NC3Rs). Renal IR was induced as described previously with small alterations^26^. The renal pedicles were clamped for 25 minutes (uni- or bilaterally) through an abdominal incision under 2.5% isoflurane-induced anaesthesia. Mice received an injection of 50 μg/kg buprenorphin (Shering-Plough) for analgesic purposes. Sham mice underwent the same procedure without clamping of the renal pedicles. Successful reperfusion was validated by regain of the initial color of the kidney after clamp removal. Methods were carried out in accordance with the relevant guidelines and regulations.

### TREM-1 inhibitors

We used 3 previously validated approaches to modulate TREM-1 function^12^,^13^,^40^. LP17 (LQVTDSGLYRCVIYHPP), LR12 (LQEEDTGEYGCV) and LP17/LR12–scramble protein (TDSRCVIGLYHPPLQVY/YQDVELCETGED) were chemically synthesized (Pepscan) based on the extracellular domain of TREM-1 and TREM-like transcript 1 (TLT1) respectively. TREM-1 fusion protein (Fc-TREM1) and control IgG were purchased from R&D. Endotoxin-free TREM-1 inhibitors or respective controls were dissolved in sterile NaCl 0.9%. WT mice received an intraperitoneal (i.p.) injection (100 μl) of Fc-TREM1 fusion protein (1, 2.5 and 5 μg) or matching isotype control, 1 hour before surgery. For LR12 experiment mice received 200 μg of LR12/control protein (100 μl total volume) i.p., starting 2 hours before the surgery and every 8 hours until the sacrifice (24 hours). Sham mice received the same treatment with scramble peptide/LR12. In the LP17 treatment, mice received different doses (50, 100 and 200 μg) of LP17 or scramble protein, 1 hour before surgery through an i.p. injection (100 μl). Mice were sacrificed 24 hours after reperfusion.

### Biological parameters related to murine kidney function, damage and inflammation

Murine renal function in the bilateral experiment was determined by plasma urea and creatinine as described before^26^. The degree of tubular damage was assessed on periodic acid-Schiff after diastase treatment (PAS-D)-stained tissue sections The PAS-D score was obtained by a nephropathologist in a blinded fashion as previously described^26^. For granulocyte staining, we used FITC-labeled anti-mouse Ly6G mAb (BD Biosciences) followed by appropriate secondary antibodies as described before^26^. Ly6G positive cells were counted in 10 high power fields (HPF). Tubular apoptosis was detected with staining for cleaved caspase-3 (Cell signalling) as previously described^26^. Positive nuclei within the tubular cells were counted in 10 HPF.

### ELISA and western blot

Circulating level of sTREM-1 were measured in plasma by ELISA (R&D). Renal levels of sTREM-1, KC, MCP-1, IL-6 and IL-1β (R&D) were measured in snap-frozen and lysed kidney homogenates (300 mM NaCl, 15 mM Tris, 2 mM MgCl, 1 mM CaCl_2_ and 1% Triton X100, pH 7.4 with 100 μg/ml pepstatin A, leupeptin and aprotinin mix)^26^. Protein levels in renal tissue were corrected for total protein level using BioRad protein assay (Bio-Rad). For western blots, kidney lysates were prepared from 10 frozen sections (20 μm thick), incubated at 4 °C for 30 min in RIPA buffer containing 50 mM Tris pH7.5, 0.15 M NaCl, 2 mM EDTA, 1% deoxycholic acid, 1% NP-40, 4 mM sodium orthovanadate, 10 mM sodium fluoride, 1% protease inhibitor cocktail (P8340, Sigma). The lysates were then centrifuged at 14000 rpm for 10 minutes and the supernatants were collected and stored at −20 °C. SDS-polyacrylamide gel electrophoresis was carried out in 4–12% gradient gels (Invitrogen) and proteins were electrophoretically transferred onto methanol-activated polyvinylidene fluoride (PVDF) microporous membranes (Millipore). Membrane was blocked for one hour with 5% milk in Tris-buffered saline containing 0.1% Tween 20 (TBS-T), followed by overnight incubation at 4 °C with primary rabbit antibody anti-TREM-1 (ab104413, 1:1500, Abcam). HRP-conjugated secondary antibodies (DAKO) were incubated for two hours at room temperature, and HRP activity was visualized with ECL-reagent (Amersham Pharmacia Biotech). Tubulin was used as loading control. Densitometric quantification analysis was performed on imagines of scanned films using the image processing program ImageJ (NIH, US).

### Flow cytometry

Peripheral blood was drawn via a cardiac puncture and red blood cells were lysed (Erythrocyte lysis solution: 160 mM NH_4_Cl, 10 mM KHCO_3_ and 0.1 mM EDTA, pH 7.4). Cell suspensions were stained with FITC-labeled anti-mouse Ly6C/Gr-1 (BD Pharmingen) and Alexa Fluor 647-labeled anti mouse F4/80 (eBioscience) to determine granulocytes and monocyte populations. PE-labeled rat anti-mouse TREM-1 (R&D) was used to determine TREM-1 positive cells within the identified leukocyte populations. Stained cells were acquired on FACS Calibur (BD Biosciences). Data analysis was performed using FlowJo v10 (FlowJo LLC).

### mRNA purification and reverse transcriptase PCR

Total RNA was isolated from 10–15 renal frozen tissue slides (20 μM) using Trizol reagent (Invitrogen) according to manufacturer’s protocol. mRNA samples were converted to cDNA (complementary DNA) using oligo-dT. Tata box-binding protein (*Tbp)*, *Trem1*, *Cxcl1*, *Ccl2*, *Il6*, *Il1b*, *Tnfa and Myd88* mRNA expression was analyzed by reverse transcriptase PCR with SYBR green PCR master mix on a Light Cycler 480 (Hoffmann-La Roche). Relative expression was analyzed using LinRegPCR (developed by Hearth failure research center, University of Amsterdam). Gene expression of *Trem1* was normalized to housekeeping gene (*Tbp*). Primer sequences are *Trem1* forward: 5′-GCGTCCCATCCTTATTACCA, reverse: 5′-AAACCAGGCTCTTGCTGAGA; *Tbp* forward: 5′-GGAGAATCATGGACCAGAACA, reverse: 5′-GATGGGAATTCCAGGAGTCA; *Cxcl1* forward: 5′ATAATGGGCTTTTACATTCTTTAACC, reverse: 5′-AGTCCTTTGAACGTCTCTGTCC; *Ccl2* forward: 5′-CATCCACGTGTTGGCTCA, reverse: 5′-GATCATCTTGCTGGTGAATGAGT; *Myd88* forward: 5′-TGGCCTTGTTAGACCGTGA; reverse: 5′-AAGTATTTCTGGCAGTCCTCCTC; *Il1b* forward: 5′-CTGCAGCTGGAGAGTGTGGAT; reverse: 5′-GCTTGTGCTCTGCTTGTGAG; *Il6* forward: 5′-GCTACCAAACTGGATATAATGGA; reverse: 5′-CCAGGTAGCTATGGTACT6CCAGAA; *Tnfa* forward: 5′-CTGTAGCCCACGTCGTAGC; reverse: 5′-TTGACATCCATGCCGTTG.

### *Trem1 in situ* hybridization

*In situ* hybridization was performed on frozen tissue sections as described before^15^,^52^ with minor adjustments. A DNA template was generated by nested PCR incorporation of T7 RNA polymerase promoters using *mTrem1* primers (forward: 5′-GCGTGTTCTTTGTCTCAGAAGT and reverse 5′-taatacgactcactataggg AGGAGAGGAAACAACCGCAG). Kidneys were perfused with PBS, fixated in 4% PFA and placed in 30% sucrose overnight at 4 °C. The next day, the tissues was placed in O.C.T. compound (Tissue-Tek). The kidney tissue sections (5 μm) were permeabilised with proteinase K (10 μg/ml), fixated and next acetylated. Riboprobe concentration of 0.5 μg/ml was used for hybridization. For riboprobe detection, sections were pre-treated with blocking buffer (20% heat inactivated sheep serum, 2% blocking reagent; Hoffmann-La Roche) and incubated with anti–DIG-AP antibody (Hoffmann-La Roche) at 4 °C overnight. The next day, a chromogenic substrate (BM Purple; Hoffmann-La Roche) was used to visualize the signal. Sections were fixated in 4% PFA and mounted with Glycergel (Dako).

### Renal transplant study population

We included samples from the Renal Genetics Transplantation (REGaTTA) cohort collected from the University Medical Center Groningen, Groningen, The Netherlands^25^. Matched donors and recipients from 1430 transplantations were assessed for eligibility. Patients with more than two re-transplantations, simultaneous kidney/pancreas- or kidney/liver transplantations, unavailability of DNA for genotyping, technical problems or patients that were lost to follow-up, were excluded. The final cohort on which statistical analyses was performed comprised of 1263 donor-recipient pairs. A detailed flow diagram is shown in Supplementary Fig. S5. The medical ethics committee of the University Medical Center Groningen approved the study under file n° METc 2014/077 and written informed consent was acquired from all living transplant donors. By Dutch jurisdiction, deceased donors provide informed consent upon registration of their donation status. No living donors from a vulnerable population were used. The study was conducted according to the Declarations of Helsinki and Istanbul.

### DNA isolation and *TREM1* variant selection

Peripheral blood mononuclear cells were used to acquire donor and recipient DNA. Based on a 1000 Genomes minor allele frequency (MAF) of >1%, 2 non-synonymous SNVs in the *TREM1* gene were selected: rs2234237 (p.Thr25Ser) and rs2234245 (p. Phe214Leu). Genotyping of the SNVs was performed using the Illumina VeraCode GoldenGate Assay kit (Illumina) according to the manufacturer’s instructions. Genotype clustering and calling were performed using BeadStudio Data Analysis Software (Illumina).

### Renal transplantation study outcomes

The primary outcome used in this study was DGF, defined as the requirement for dialysis within the first week after transplantation (patients with primary non-function of the graft^53^, were excluded, since these cases most probably represent arterial and venous thrombosis instead of IR). Secondary outcomes were time-to-first episode of biopsy-proven acute rejection (BPAR) and death-censored graft survival (DCGF: defined as the need for indefinite dialysis or re-transplantation).

### Statistical analyses

In the murine studies, differences between groups were analysed using non-parametric Mann-Whitney (2 groups) or Kruskal-Wallis tests (>2 groups) followed by Mann Whitney (treatment compared to control) using Prism (GraphPad Software). Results are expressed as mean ± SEM. Values of P < 0.05 were considered significant. For the human renal transplantation study, deviation from Hardy-Weinberg equilibria were tested with PLINK^54^. Differences in allele frequencies between donors and recipients were tested by logistic regression analyses in a genetic additive model construction. Results were adjusted for age and donor-recipient relatedness with the DFAM algorithm. Minor allele frequencies between the HapMap-CEU (a population from Utah with Northern and Western European ancestry) that was genotyped in the context of the 1000 Genomes study were compared by Fisher exact tests or χ^2^ tests where appropriate. The association between *TREM1* SNVs and DGF were tested with logistic regression models with stratification for donor type. The association with biopsy-proven acute rejection and death-censored graft survival was tested with Cox proportional hazard models. An insufficient amount of patients had a homozygous recessive genotype and we therefore only calculated odds (OR) and hazard (HR) ratios for the heterozygous genotype. Two-sided *P*-values below 0.05 after Bonferroni correction were considered significant. Data were analyzed with the R computing environment.

## Additional Information

**How to cite this article**: Tammaro, A. *et al*. Effect of TREM-1 blockade and single nucleotide variants in experimental renal injury and kidney transplantation. *Sci. Rep.* **6**, 38275; doi: 10.1038/srep38275 (2016).

**Publisher's note:** Springer Nature remains neutral with regard to jurisdictional claims in published maps and institutional affiliations.

## Supplementary Material

Supplementary Information

## Figures and Tables

**Table 1:** No differences in cytokines and chemokine protein in kidney homogenates of mice treated with different TREM-1 inhibitors.

Cytokine fraction	Treatment			
		Fc-TREM-1		
	Control	1 μg	2.5 μg	5 μg
KC (pg/mg)	407 ± 19	443 ± 64	400 ± 123	259 ± 78
MCP-1 (pg/mg)	242 ± 45	176 ± 21	132 ± 16	152 ± 18
IL-6 (pg/mg)	27 ± 2	29 ± 4	21 ± 2	27 ± 3
IL-1β (pg/mg)	31 ± 3	38 ± 6	31 ± 4	43 ± 9
	**Scramble**	**LR12**	**Scramble**	**LR12**
	**Sham**	**Sham**	**IR**	**IR**
KC (pg/mg)	45 ± 7	48 ± 4	251 ± 64^$^	200 ± 40^&^
MCP-1 (pg/mg)	280 ± 16	301 ± 27	284 ± 24	249 ± 20
IL-6 (pg/mg	78 ± 10	79 ± 6	91 ± 7	91 ± 4
IL-1β (pg/mg)	86 ± 5	92 ± 6	172 ± 55^#^	86 ± 7^@^
		**LP17**		
	**Control**	**50 μg**	**100 μg**	**200 μg**
KC (pg/mg)	407 ± 19	373 ± 63	447 ± 38	390 ± 23
MCP-1 (pg/mg)	267 ± 44	189 ± 14	411 ± 120	164 ± 58
IL-6 (pg/mg)	27 ± 2	40 ± 1	74 ± 22	39 ± 3
IL-1β (pg/mg)	31 ± 3	46 ± 4	127 ± 69	40 ± 1

Data are presented as mean ± SEM and analysed with non-parametric tests. Fc-TREM1 (n = 4), LR12 (n = 8) and LP17 (n = 4). ^$^P = 0.001 versus sham scramble. ^&^P = 0.0007 versus sham LR12. ^#^P = 0.01 versus sham LR12. ^@^P = 0.02 versus IR scramble. KC, keratinocyte-derived chemokine ( =  CXCL-1, chemokine C-X-C motif ligand 1); MCP-1, monocyte chemoattractant protein 1 (=CCL-2, chemokine C-C motif ligand 2); IL-6, interleukin 6; IL-1β, interleukin 1β.

**Table 2:** Characteristics of study groups.

Parameter	Value
N	1263
**Donor characteristics**	
Age (mean years ± SE)	44 ± 14
Male N (%)	645 (51%)
Donortype N (%)	
Cadaveric donor	989 (78%)
−DBD	787 (62%)
−DCD	202 (16%)
Donor cause of death N (%)	
−CVA	549 (43%)
−Trauma	305 (24%)
−Other	135 (11%)
−Unknown	282 (22%)
**Recipient characteristics**	
Age (mean years ± SE)	48 ± 14
Male N (%)	739 (58%)
Primary kidney disease N (%)	
−Glomerulonephritis	271 (21%)
−Adult polycyctic kidney disease	167 (13%)
−Renal vascular disease	124 (10%)
−IgA nephropathy	98 (8%)
−Pyelonephritis	148 (12%)
−Diabetic nephropathy	51 (4%)
−End-stage renal disease with unknown etiology	168 (13%)
−Other	244 (19%)
Initial immunosuppression N (%)	
−Corticosteroids	1201 (95%)
−Mycophenolic acid	907 (71%)
−Cyclosporine A	1085 (85%)
Azithioprine	72 (6%)
−Tacrolimus	97 (8%)
−Sirolimus	38 (3%)
Induction therapy N (%)	103 (8%)
−ATG	19 (2%)
−Anti-CD3 moab	199 (16%)
−Interleukin-2 RA	
First renal transplant N (%)	1142 (90%)
**Transplant characteristics**	
Cold ischemia time (mean hours ± SE)	
−Living donor	3 ± 2
−Cadaveric donor	21 ± 7
HLA no. of 0 mismatches N (%)	241/1050 (23%)

DBD = deceased brain death, DCD = deceased cardiac death, CVA = cerebrovasculair accident, ATG = antithymocyte globulin RA = receptor antagonist, SE = standard error.

**Table 3:** Donor and recipient genotype distribution of *TREM1* single nucleotide variants.

HGVS (rs number)	Phenotype (SNVinfo)	A/a	1000Genomes	Donor^1^				Recipient^1^				*P*^2^
			MAF	A/A	A/a	a/a	MAF	A/A	A/a	a/a	MAF	
p.Thr25Ser (rs2234237)	Unknown (benign^5^)	A/t	**0.080**^3^	0.845	0.155	0.000	**0.078**	0.836	0.159	0.005	**0.085**	0.8
p.Phe214Leu (rs2234245)	Unknown (benign^5^)	G/c	**0.028**^4^	0.999	0.001	0.000	**0.001**	1.000	0.000	0.000	**0.000**	1

^1^Donor and recipient genotype are displayed as homyzgous dominant (A/A), heterozygous (A/a) or homozygous recessive (a/a). MAF = minor allele frequency.

^2^P-value for logistic regression between donor and recipient allele frequencies in a genetic additive model, adjusted for age and donor-recipient relatedness (DFAM algorithm).

^3^HapMap-CEU population.

^4^Overall 1000 Genomes population.

^5^Predicted benign by Polyphen (most likely lacking a phenotypical effect).

**Table 4:** Association of *TREM1* single nucleotide variant p.Thr25Ser with renal outcomes.

	Donor		Recipient	
Delayed graft function				
Cohort	OR_heterozygosity_ (95% CI)	*P*-value^1^	OR_heterozygosity_ (95% CI)	*P*-value^1^
Full	1.08 (0.77–1.52)	1	0.91 (0.64–1.27)	1
DBD	1.07 (0.68–1.65)	1	1.03 (0.66–1.59)	1
DCD	0.77 (0.31–2.11)	1	0.69 (0.28–1.90)	1
**Biopsy-proven acute rejection**				
	**HR_heterozygosity_ (95% CI)**	***P*-value^1^**	**HR_heterozygosity_ (95% CI)**	***P*-value^1^**
Full	0.72 (0.54–0.96)	0.15	0.81 (0.62–1.07)	0.84
DBD	0.70 (0.49–1.00)	0.29	0.86 (0.62–1.20)	1
DCD	1.10 (0.56–2.16)	1	1.04 (0.53–2.04)	1
**Death-censored graft failure**				
Full	0.75 (0.50–1.13)	1	0.87 (0.59–1.27)	1
DBD	0.89 (0.57–1.41)	1	0.84 (0.53–1.34)	1
DCD	0.50 (0.18–1.39)	1	1.18 (0.52–2.64)	1

^1^After Bonferroni correction. OR_heterozygosity_ = odds ratio (homozygous recessive were not sufficiently represented for statistical analyses), CI = confidence interval. DBD = deceased brain death donor, DCD = deceased cardiac death donor.
