# Corrigendum: Effect of TREM-1 blockade and single nucleotide variants in experimental renal injury and kidney transplantation

**DOI:** 10.1038/srep44163

**Published:** 2017-03-15

**Authors:** Alessandra Tammaro, Jesper Kers, Diba Emal, Ingrid Stroo, Gwendoline J. D. Teske, Loes M. Butter, Nike Claessen, Jeffrey Damman, Marc Derive, Gerjan J. Navis, Sandrine Florquin, Jaklien C. Leemans, Mark C. Dessing

Scientific Reports 6: Article number: 3827510.1038/srep38275; published online: 12 08 2016; updated: 03 15 2017

The original version of this Article contained errors in the spelling of the authors Alessandra Tammaro, Jesper Kers, Diba Emal, Ingrid Stroo, Gwendoline J. D. Teske, Loes M. Butter, Nike Claessen, Jeffrey Damman, Marc Derive, Gerjan J. Navis, Sandrine Florquin, Jaklien C. Leemans and Mark C. Dessing which were incorrectly given as A. Tammaro, J. Kers, D. Emal, I. Stroo, G. J. D. Teske, L. M. Butter, N. Claessen, J. Damman, M. Derive, G. Navis, S. Florquin, J. C. Leemans and M. C. Dessing.

This has now been corrected in the PDF and HTML versions of the Article.

