# Supplementary material for: Effect of TREM-1 blockade and single nucleotide variants in experimental renal injury and kidney transplantation
Source: Sci Rep. 2016 Dec 8;6:38275. doi: 10.1038/srep38275 (PMC5143803; doi:10.1038/srep38275)
Supplement: Supplementary Information [file srep38275-s1.pdf]

Supplementary Information

Effect of TREM-1 blockade and single nucleotide variants in experimental renal injury and kidney transplantation

Alessandra Tammaro[1,ζ]*, Jesper Kers[1 ζ], Diba Emal[1], Ingrid Stroo[1], Gwen J. D. Teske[1], Loes M. Butter[1], Nike Claessen[1], Jeffrey Damman[1], Marc Derive[2], Gerjan J. Navis[3,5], Sandrine Florquin[1,4], Jaklien C. Leemans[1], Mark C. Dessing[1]

[1]Department of Pathology, Academic Medical Center, University of Amsterdam, Amsterdam, The Netherlands. [2]INSERM UMR_S1116, Faculté de Médecine de Nancy, Université de Lorraine, Vandœuvre-les-Nancy, France. [3]Department of Internal Medicine, Division of Nephrology, University Medical Center Groningen, University of Groningen, Groningen, The Netherlands. [4]Department of Pathology, Radboud University Nijmegen Medical Center, Nijmegen, The Netherlands. [5]On behalf of the REGaTTA (REnal GeneTics TrAnsplantation) Groningen group. [ζ] These authors contributed equally to this work. *Correspondence and requests for materials should be addressed to A.T. (email: a.tammaro@amc.uva.nl)

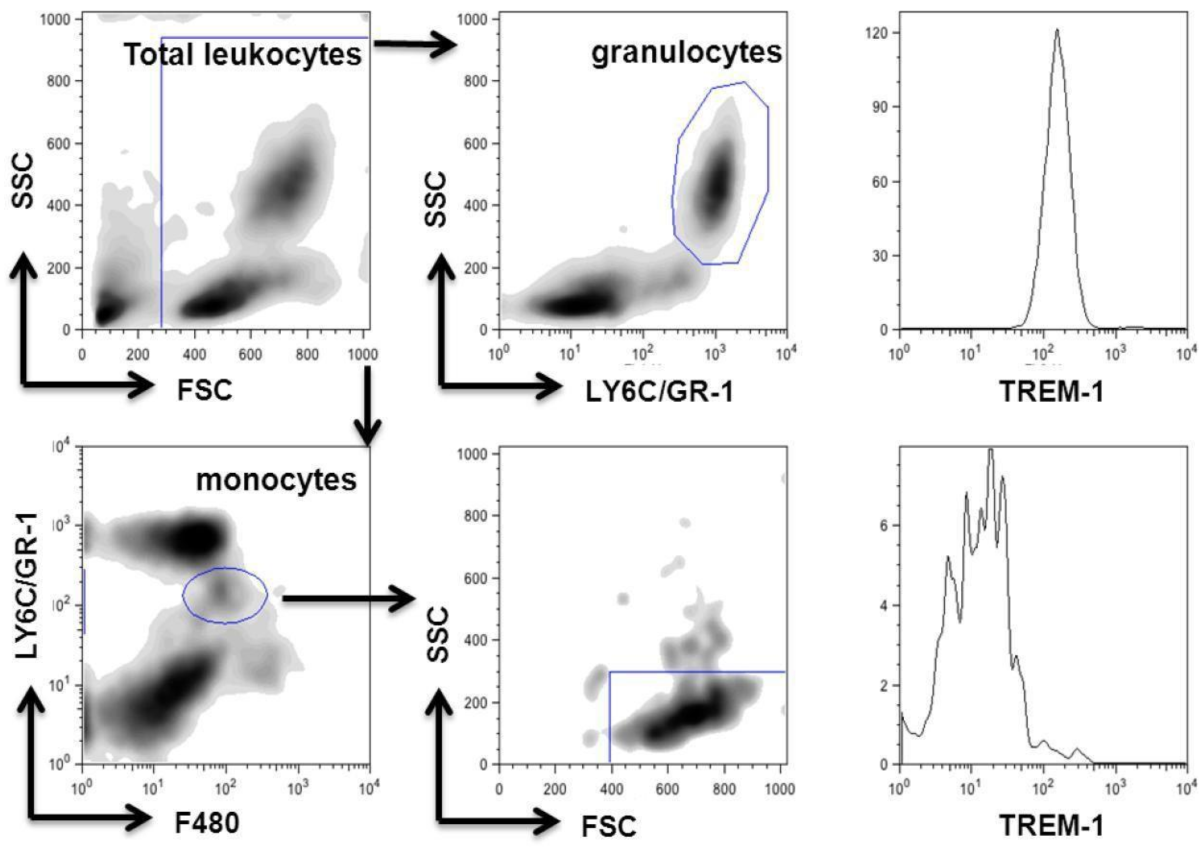

Supplementary Figure S1: Gating strategy of circulating leukocytes. Granulocytes are shown as percentage of LY6C/Gr-1 high by SSC of total leukocytes. Cells in the monocytes gate were identified as Ly6C/Gr-1positive and F4-80-negative (excluding granulocyte population by forward and side scatter). TREM-1 staining on each population is shown by histograms.

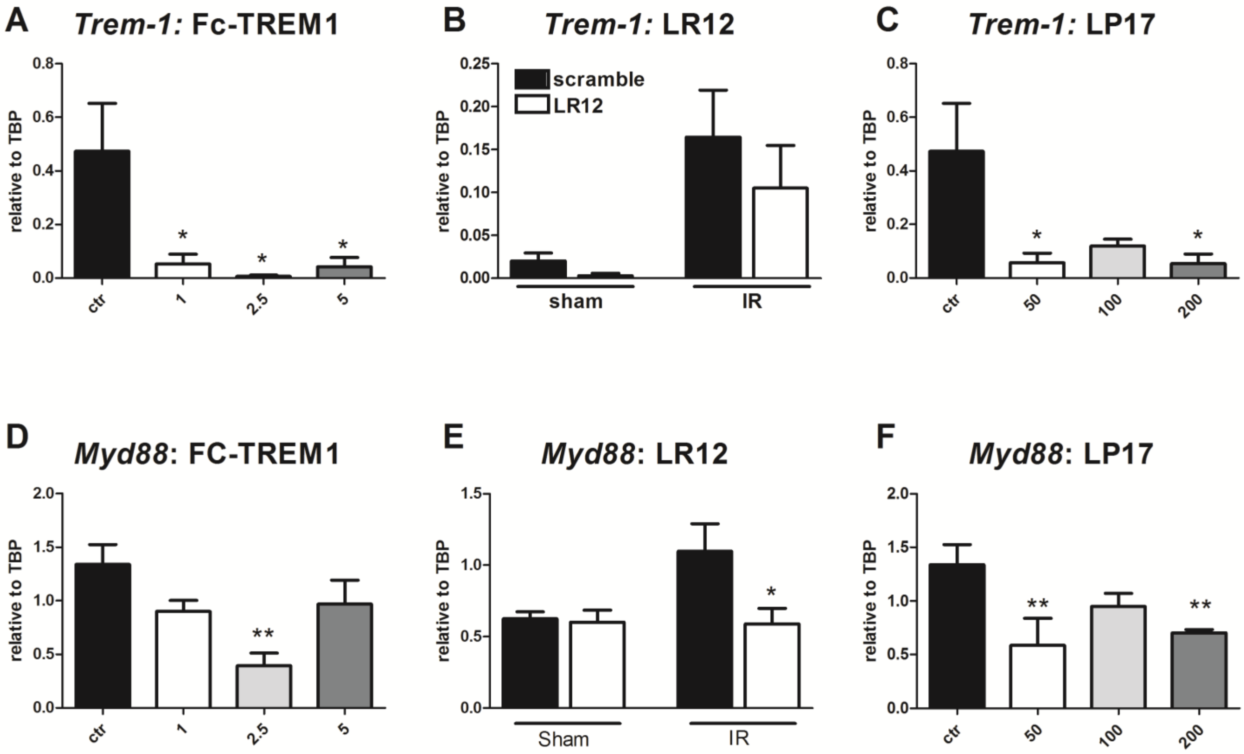

Supplementary Figure S2. TREM-1 expression and pathway are down-regulated by different inhibitors. *Trem1* and *Myd88* mRNA expression as measured by RT-PCR in the groups treated with Fc-TREM1 (**A,D**) (n = 4/group), LR12 (**B,E**) (n = 6-8/group) and LP17 (**C,F**) (n = 4/group). Data are normalized to *Tbp* expression. Values are expressed as mean ± SEM. *P < 0.05 vs control. **P < 0.005 versus control.

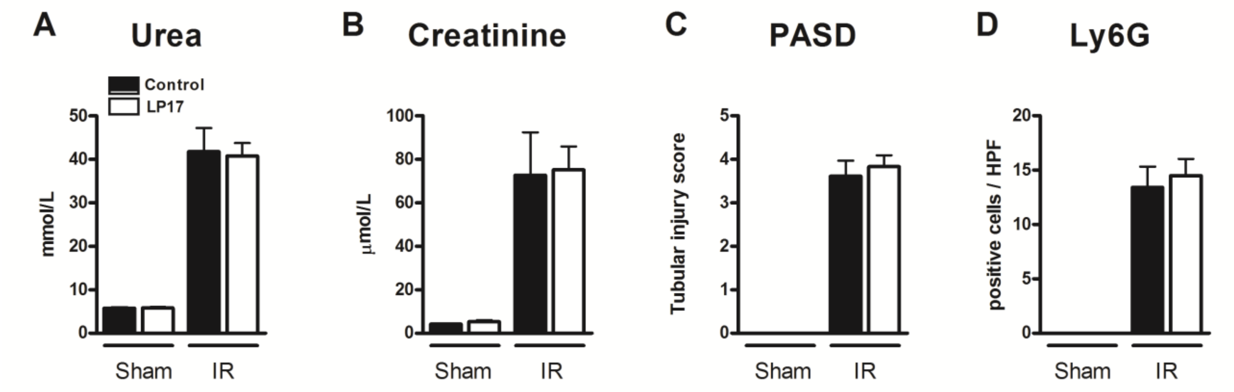

Supplementary Figure S3. LP17 pre-treatment does not prevent IR-induced injury in bilateral renal IR experiment. Plasma urea (**A**), creatinine (**B**), renal damage (**C**) and granulocyte influx (**D**) in mice pre-treated with 200 µg of LP17 or control protein in bilateral ischemia model. Treatment was performed 1 hour before surgery. Mice received 100 µl of LP17/control protein dissolved in sterile NaCl by intra-peritoneal injection. Sham mice received the same treatment (n =8-10/group).

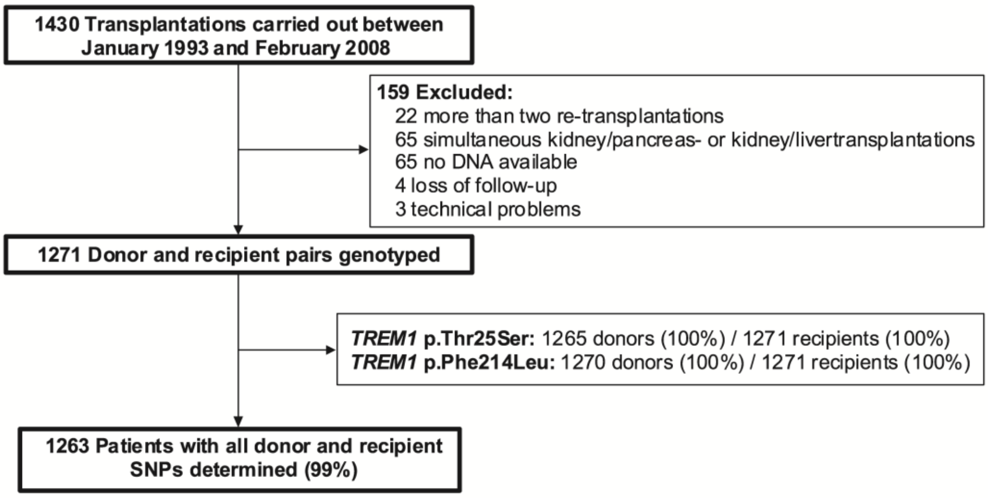

Supplementary Figure S4. Flow diagram of the in- and excluded patients with the amount of missing data on the three single nucleotide variants in donors and recipients.

Pt	Age (yr)	Donor type	CIT (min)	WIT2 (min)	DGF	Rejection	Time to rejection (months)	DCGF	Time to DCGF (years)
1	64	Deceased	1800	30	No	No	-	No	-
2	36	Deceased	720	17	No	Yes	1	No	-
3	59	Deceased	1560	35	No	Yes	1	Yes	7
4	35	Deceased	115	37	No	Yes	1	No	-
5	24	Deceased	152	41	No	Yes	5	Yes	2

Supplementary Table S1. Characteristics of the 5 renal transplant recipients with a homozygous recessive variant of *TREM1* p.Thr25Ser. Pt, patient; CIT, cold ischemia time; WIT2, anastomosis time; DGF, delayed graft function; DCGF, death-censored graft failure.
